# 5-Cyclo­pentyl-2-(4-fluoro­phen­yl)-3-isopropyl­sulfonyl-1-benzofuran

**DOI:** 10.1107/S1600536812045916

**Published:** 2012-11-10

**Authors:** Hong Dae Choi, Pil Ja Seo, Uk Lee

**Affiliations:** a Department of Chemistry, Dongeui University, San 24 Kaya-dong, Busanjin-gu, Busan 614-714, Republic of Korea; b Department of Chemistry, Pukyong National University, 599-1 Daeyeon 3-dong, Nam-gu, Busan 608-737, Republic of Korea

## Abstract

In the title compound, C_22_H_23_FO_3_S, the cyclo­pentyl ring adopts an envelope conformation with the flap atom connected to the benzofuran residue. The 4-fluoro­phenyl ring makes a dihedral angle of 43.67 (3)° with the mean plane [r.m.s. deviation = 0.008 (1) Å] of the benzofuran fragment. In the crystal, mol­ecules are linked by weak C—H⋯O and C—H⋯π inter­actions, forming a three--dimensional network. The crystal structure also exhibits slipped π–π inter­actions between the benzene and furan rings of neighbouring mol­ecules [centroid–centroid distance = 3.883 (2) Å and slippage = 1.731 (2) Å].

## Related literature

For background information and the crystal structures of related compounds, see: Choi *et al.* (2011 ▶); Seo *et al.* (2011 ▶).
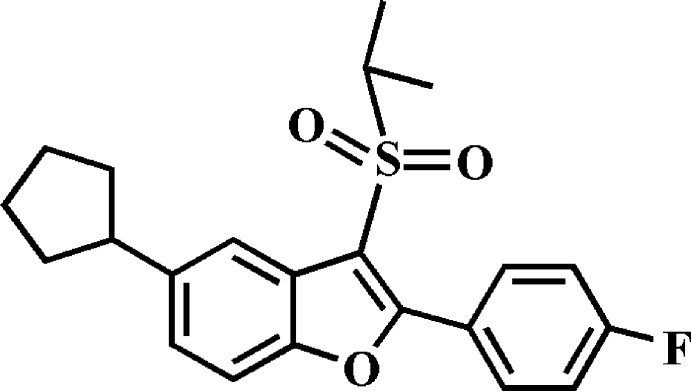


## Experimental

### 

#### Crystal data

C_22_H_23_FO_3_S
*M* *_r_* = 386.46
Monoclinic, 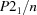

*a* = 9.4736 (3) Å
*b* = 19.6185 (7) Å
*c* = 10.8018 (3) Å
β = 108.833 (1)°
*V* = 1900.12 (10) Å^3^
*Z* = 4
Mo *K*α radiation
μ = 0.20 mm^−1^
*T* = 173 K
0.40 × 0.39 × 0.36 mm

#### Data collection

Bruker SMART APEXII CCD diffractometer
Absorption correction: multi-scan (*SADABS*; Bruker, 2009 ▶) *T*_min_ = 0.678, *T*_max_ = 0.746
18610 measured reflections
4714 independent reflections
3948 reflections with *I* > 2σ(*I*)
*R*_int_ = 0.030

#### Refinement

*R*[*F* ^2^ > 2σ(*F* ^2^)] = 0.040
*wR*(*F* ^2^) = 0.103
*S* = 1.03
4714 reflections
246 parameters
H-atom parameters constrained
Δρ_max_ = 0.40 e Å^−3^
Δρ_min_ = −0.35 e Å^−3^

### 

Data collection: *APEX2* (Bruker, 2009 ▶); cell refinement: *SAINT* (Bruker, 2009 ▶); data reduction: *SAINT*; program(s) used to solve structure: *SHELXS97* (Sheldrick, 2008 ▶); program(s) used to refine structure: *SHELXL97* (Sheldrick, 2008 ▶); molecular graphics: *ORTEP-3* (Farrugia, 2012 ▶) and *DIAMOND* (Brandenburg, 1998 ▶); software used to prepare material for publication: *SHELXL97*.

## Supplementary Material

Crystal structure: contains datablock(s) global, I. DOI: 10.1107/S1600536812045916/fy2075sup1.cif

Structure factors: contains datablock(s) I. DOI: 10.1107/S1600536812045916/fy2075Isup2.hkl

Supplementary material file. DOI: 10.1107/S1600536812045916/fy2075Isup3.cml

Additional supplementary materials:  crystallographic information; 3D view; checkCIF report

## Figures and Tables

**Table 1:** Hydrogen-bond geometry (Å, °) *Cg*1 is the centroid of the C2–C7 benzene ring.

*D*—H⋯*A*	*D*—H	H⋯*A*	*D*⋯*A*	*D*—H⋯*A*
C6—H6⋯O2^i^	0.95	2.60	3.3341 (17)	135
C18—H18⋯O3^ii^	0.95	2.48	3.2932 (19)	144
C21—H21*A*⋯*Cg*1^iii^	0.98	2.71	3.693 (2)	177

Symmetry codes: (i) 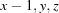; (ii) 
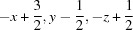
; (iii) 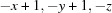.

**Fig. 1.**
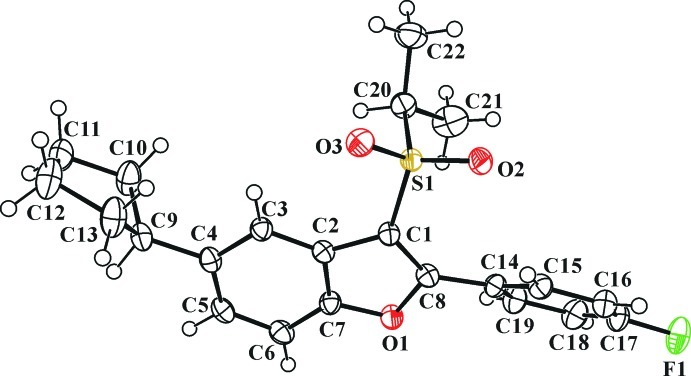
The molecular structure of the title compound with the atom numbering scheme. Displacement ellipsoids are drawn at the 50% probability level. H atoms are presented as small spheres of arbitrary radius.

**Fig. 2.**
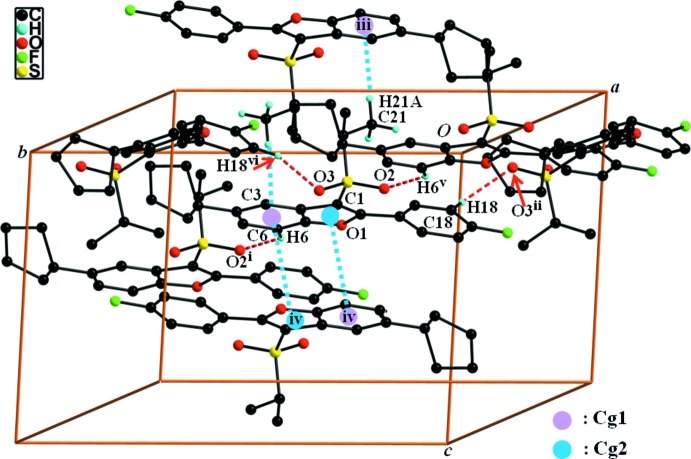
A view of the C—H···O, C—H···π and π–π interactions (dotted lines) in the crystal structure of the title compound. H atoms non-participating in hydrogen-bonding were omitted for clarity. [Symmetry codes: (i) x -1, y, z; (ii) - x + 1.5, y - 0.5, - z + 0.5; (iii) - x + 1, - y + 1, - z; (iv) - x + 1, - y + 1, - z + 1; (v) x + 1, y, z; (vi) - x + 1/2, y + 1/2, - z + 1/2.]

## supplementary crystallographic information

### Comment

As a part of our ongoing study of 5-cyclopentyl-1-benzofuran derivatives containing (2-phenyl-3-methylsulfinyl) (Choi *et al.*, 2011) and {2-(4-fluorophenyl)-3-methylsulfinyl} (Seo *et al.*, 2011) substituents, we report herein the crystal structure of the title compound.

In the title molecule (Fig. 1), the benzofuran unit is essentially planar, with a mean deviation of 0.008 (1) Å from the least-squares plane defined by the nine constituent atoms. The cyclopentyl ring has an envelope conformation. The dihedral angle between the 4-fluorophenyl ring and the mean plane of the benzofuran fragment is 43.67 (3)°. In the crystal structure (Fig. 2), molecules are connected by weak C—H···O and C—H···π interactions (Table 1, Cg1 is the centroid of the C2–C7 benzene ring). The crystal packing (Fig. 2) also exhibits slipped π–π interactions between the benzene and furan rings of neighbouring molecules, with a Cg1···Cg2^iv^ distance of 3.883 (2) Å and an interplanar distance of 3.476 (2) Å resulting in a slippage of 1.731 (2) Å (Cg2 is the centroid of the C1/C2/C7/O1/C8 furan ring, iv: -*x* + 1, -*y* + 1, -*z* + 1).

### Experimental

3-Chloroperoxybenzoic acid (77%, 381 mg, 1.7 mmol) was added in small portions to a stirred solution of 5-cyclopentyl-2-(4-fluorophenyl)-3-isopropylsulfanyl-1-benzofuran (283 mg, 0.8 mmol) in dichloromethane (40 mL) at 273 K. After being stirred at room temperature for 10h, the mixture was washed with saturated sodium bicarbonate solution and the organic layer was separated, dried over magnesium sulfate, filtered and concentrated at reduced pressure. The residue was purified by column chromatography (benzene) to afford the title compound as a colorless solid [yield 67%, m.p. 397–398 K; Rf = 0.62 (benzene)]. Single crystals suitable for X-ray diffraction were prepared by slow evaporation of a solution of the title compound in benzene at room temperature.

### Refinement

All H atoms were geometrically positioned and refined using a riding model, with C—H = 0.95 Å for aryl, 1.00 Å for methine, 0.99 Å for methylene and 0.98 Å for methyl H atoms, respectively. *U*_iso_(H) = 1.2*U*_eq_(C) for aryl, methine, methylene and 1.5*U*_eq_(C) for methyl H atoms. The positions of methyl hydrogens were optimized rotationally.

### Crystal data

**Table:**

C_22_H_23_FO_3_S	*F*(000) = 816
*M**_r_* = 386.46	*D*_x_ = 1.351 Mg m^−^^3^
Monoclinic, *P*2_1_/*n*	Melting point = 397–398 K
Hall symbol: -P 2yn	Mo *K*α radiation, λ = 0.71073 Å
*a* = 9.4736 (3) Å	Cell parameters from 5864 reflections
*b* = 19.6185 (7) Å	θ = 2.3–28.0°
*c* = 10.8018 (3) Å	µ = 0.20 mm^−^^1^
β = 108.833 (1)°	*T* = 173 K
*V* = 1900.12 (10) Å^3^	Block, colourless
*Z* = 4	0.40 × 0.39 × 0.36 mm

### Data collection

**Table:**

Bruker SMART APEXII CCD diffractometer	4714 independent reflections
Radiation source: rotating anode	3948 reflections with *I* > 2σ(*I*)
Graphite multilayer monochromator	*R*_int_ = 0.030
Detector resolution: 10.0 pixels mm^-1^	θ_max_ = 28.3°, θ_min_ = 2.1°
φ and ω scans	*h* = −12→12
Absorption correction: multi-scan (*SADABS*; Bruker, 2009)	*k* = −22→26
*T*_min_ = 0.678, *T*_max_ = 0.746	*l* = −14→14
18610 measured reflections	

### Refinement

**Table:**

Refinement on *F*^2^	Primary atom site location: structure-invariant direct methods
Least-squares matrix: full	Secondary atom site location: difference Fourier map
*R*[*F*^2^ > 2σ(*F*^2^)] = 0.040	Hydrogen site location: difference Fourier map
*wR*(*F*^2^) = 0.103	H-atom parameters constrained
*S* = 1.03	*w* = 1/[σ^2^(*F*_o_^2^) + (0.0509*P*)^2^ + 0.6277*P*] where *P* = (*F*_o_^2^ + 2*F*_c_^2^)/3
4714 reflections	(Δ/σ)_max_ = 0.001
246 parameters	Δρ_max_ = 0.40 e Å^−^^3^
0 restraints	Δρ_min_ = −0.35 e Å^−^^3^

### Special details

**Table:**

Geometry. All esds (except the esd in the dihedral angle between two l.s. planes) are estimated using the full covariance matrix. The cell esds are taken into account individually in the estimation of esds in distances, angles and torsion angles; correlations between esds in cell parameters are only used when they are defined by crystal symmetry. An approximate (isotropic) treatment of cell esds is used for estimating esds involving l.s. planes.

### Fractional atomic coordinates and isotropic or equivalent isotropic displacement parameters (Å^2^)

**Table:**

	*x*	*y*	*z*	*U*_iso_*/*U*_eq_	
S1	0.81280 (3)	0.528906 (17)	0.27421 (3)	0.02105 (10)	
F1	0.90366 (12)	0.18165 (5)	0.46703 (11)	0.0459 (3)	
O1	0.48867 (10)	0.43110 (5)	0.35291 (10)	0.0264 (2)	
O2	0.93832 (11)	0.48394 (5)	0.32355 (11)	0.0299 (2)	
O3	0.83351 (11)	0.60006 (5)	0.30821 (10)	0.0297 (2)	
C1	0.66190 (14)	0.50030 (7)	0.31937 (13)	0.0215 (3)	
C2	0.53071 (14)	0.54131 (7)	0.30820 (13)	0.0217 (3)	
C3	0.49027 (15)	0.60966 (7)	0.28294 (13)	0.0235 (3)	
H3	0.5589	0.6419	0.2697	0.028*	
C4	0.34694 (15)	0.62964 (7)	0.27761 (13)	0.0251 (3)	
C5	0.24785 (15)	0.58109 (8)	0.29843 (14)	0.0275 (3)	
H5	0.1506	0.5955	0.2939	0.033*	
C6	0.28562 (15)	0.51320 (8)	0.32526 (14)	0.0280 (3)	
H6	0.2180	0.4809	0.3399	0.034*	
C7	0.42847 (15)	0.49561 (7)	0.32929 (14)	0.0238 (3)	
C8	0.63093 (14)	0.43500 (7)	0.34624 (13)	0.0229 (3)	
C9	0.29291 (16)	0.70217 (8)	0.24587 (15)	0.0292 (3)	
H9	0.2007	0.7076	0.2709	0.035*	
C10	0.2528 (2)	0.72112 (9)	0.10096 (16)	0.0411 (4)	
H10A	0.3331	0.7074	0.0660	0.049*	
H10B	0.1587	0.6988	0.0487	0.049*	
C11	0.2353 (2)	0.79875 (9)	0.09816 (17)	0.0416 (4)	
H11A	0.1295	0.8115	0.0807	0.050*	
H11B	0.2706	0.8190	0.0295	0.050*	
C12	0.3312 (2)	0.82331 (9)	0.2342 (2)	0.0471 (5)	
H12A	0.4107	0.8543	0.2271	0.057*	
H12B	0.2692	0.8479	0.2778	0.057*	
C13	0.3992 (2)	0.75922 (9)	0.31167 (18)	0.0424 (4)	
H13A	0.5003	0.7506	0.3070	0.051*	
H13B	0.4056	0.7639	0.4046	0.051*	
C14	0.70929 (15)	0.36969 (7)	0.37007 (14)	0.0241 (3)	
C15	0.85560 (16)	0.36436 (7)	0.45435 (14)	0.0256 (3)	
H15	0.9103	0.4044	0.4896	0.031*	
C16	0.92156 (16)	0.30085 (8)	0.48691 (15)	0.0298 (3)	
H16	1.0204	0.2966	0.5456	0.036*	
C17	0.83981 (18)	0.24418 (8)	0.43187 (16)	0.0323 (3)	
C18	0.69763 (19)	0.24717 (8)	0.34516 (16)	0.0347 (3)	
H18	0.6461	0.2069	0.3069	0.042*	
C19	0.63156 (17)	0.31065 (8)	0.31509 (15)	0.0306 (3)	
H19	0.5325	0.3141	0.2566	0.037*	
C20	0.74619 (16)	0.52264 (8)	0.09910 (14)	0.0292 (3)	
H20	0.6493	0.5477	0.0655	0.035*	
C21	0.7201 (2)	0.44906 (10)	0.05634 (18)	0.0481 (5)	
H21A	0.6878	0.4466	−0.0394	0.072*	
H21B	0.6426	0.4296	0.0878	0.072*	
H21C	0.8128	0.4233	0.0929	0.072*	
C22	0.8593 (2)	0.55731 (9)	0.04609 (17)	0.0410 (4)	
H22A	0.9560	0.5344	0.0810	0.061*	
H22B	0.8698	0.6053	0.0729	0.061*	
H22C	0.8246	0.5545	−0.0496	0.061*	

### Atomic displacement parameters (Å^2^)

**Table:**

	*U*^11^	*U*^22^	*U*^33^	*U*^12^	*U*^13^	*U*^23^
S1	0.01823 (16)	0.01971 (18)	0.02505 (17)	−0.00166 (12)	0.00676 (12)	−0.00080 (12)
F1	0.0517 (6)	0.0212 (5)	0.0596 (7)	0.0103 (4)	0.0106 (5)	0.0027 (4)
O1	0.0223 (5)	0.0215 (5)	0.0367 (6)	−0.0023 (4)	0.0113 (4)	0.0004 (4)
O2	0.0193 (5)	0.0320 (6)	0.0380 (6)	0.0034 (4)	0.0090 (4)	0.0067 (5)
O3	0.0314 (5)	0.0216 (5)	0.0373 (6)	−0.0074 (4)	0.0124 (4)	−0.0046 (4)
C1	0.0190 (6)	0.0198 (6)	0.0253 (6)	−0.0007 (5)	0.0065 (5)	−0.0015 (5)
C2	0.0196 (6)	0.0228 (7)	0.0225 (6)	−0.0011 (5)	0.0063 (5)	−0.0020 (5)
C3	0.0231 (6)	0.0216 (7)	0.0260 (7)	−0.0002 (5)	0.0082 (5)	−0.0008 (5)
C4	0.0245 (6)	0.0257 (7)	0.0244 (7)	0.0026 (5)	0.0066 (5)	−0.0035 (5)
C5	0.0199 (6)	0.0332 (8)	0.0298 (7)	0.0020 (6)	0.0087 (5)	−0.0046 (6)
C6	0.0223 (7)	0.0302 (8)	0.0329 (7)	−0.0040 (6)	0.0110 (6)	−0.0028 (6)
C7	0.0235 (6)	0.0204 (7)	0.0270 (7)	−0.0007 (5)	0.0077 (5)	−0.0010 (5)
C8	0.0202 (6)	0.0228 (7)	0.0256 (7)	−0.0025 (5)	0.0071 (5)	−0.0029 (5)
C9	0.0256 (7)	0.0268 (8)	0.0355 (8)	0.0056 (6)	0.0103 (6)	−0.0023 (6)
C10	0.0478 (10)	0.0339 (9)	0.0335 (9)	0.0123 (8)	0.0018 (7)	−0.0007 (7)
C11	0.0459 (10)	0.0343 (10)	0.0449 (10)	0.0155 (8)	0.0150 (8)	0.0075 (7)
C12	0.0444 (10)	0.0260 (9)	0.0634 (12)	0.0050 (7)	0.0070 (9)	−0.0025 (8)
C13	0.0415 (9)	0.0304 (9)	0.0444 (10)	0.0063 (7)	−0.0014 (7)	−0.0084 (7)
C14	0.0269 (7)	0.0188 (7)	0.0272 (7)	−0.0003 (5)	0.0098 (5)	0.0003 (5)
C15	0.0266 (7)	0.0218 (7)	0.0287 (7)	−0.0017 (5)	0.0092 (6)	−0.0014 (6)
C16	0.0287 (7)	0.0277 (8)	0.0321 (8)	0.0025 (6)	0.0083 (6)	0.0012 (6)
C17	0.0413 (8)	0.0188 (7)	0.0381 (8)	0.0067 (6)	0.0148 (7)	0.0020 (6)
C18	0.0412 (9)	0.0200 (8)	0.0394 (9)	−0.0042 (6)	0.0082 (7)	−0.0051 (6)
C19	0.0298 (7)	0.0233 (7)	0.0346 (8)	−0.0019 (6)	0.0047 (6)	−0.0026 (6)
C20	0.0290 (7)	0.0333 (8)	0.0239 (7)	0.0019 (6)	0.0065 (6)	−0.0006 (6)
C21	0.0667 (12)	0.0422 (11)	0.0345 (9)	−0.0129 (9)	0.0150 (8)	−0.0137 (8)
C22	0.0479 (10)	0.0459 (10)	0.0356 (9)	−0.0007 (8)	0.0225 (7)	0.0012 (7)

### Geometric parameters (Å, º)

**Table:**

S1—O2	1.4377 (10)	C11—H11A	0.9900
S1—O3	1.4406 (11)	C11—H11B	0.9900
S1—C1	1.7455 (13)	C12—C13	1.533 (2)
S1—C20	1.7946 (14)	C12—H12A	0.9900
F1—C17	1.3663 (17)	C12—H12B	0.9900
O1—C8	1.3749 (15)	C13—H13A	0.9900
O1—C7	1.3776 (17)	C13—H13B	0.9900
C1—C8	1.3661 (19)	C14—C15	1.395 (2)
C1—C2	1.4526 (18)	C14—C19	1.398 (2)
C2—C7	1.3916 (18)	C15—C16	1.387 (2)
C2—C3	1.3971 (19)	C15—H15	0.9500
C3—C4	1.3965 (18)	C16—C17	1.375 (2)
C3—H3	0.9500	C16—H16	0.9500
C4—C5	1.405 (2)	C17—C18	1.371 (2)
C4—C9	1.514 (2)	C18—C19	1.385 (2)
C5—C6	1.385 (2)	C18—H18	0.9500
C5—H5	0.9500	C19—H19	0.9500
C6—C7	1.3836 (18)	C20—C21	1.511 (2)
C6—H6	0.9500	C20—C22	1.528 (2)
C8—C14	1.4613 (19)	C20—H20	1.0000
C9—C13	1.520 (2)	C21—H21A	0.9800
C9—C10	1.533 (2)	C21—H21B	0.9800
C9—H9	1.0000	C21—H21C	0.9800
C10—C11	1.531 (2)	C22—H22A	0.9800
C10—H10A	0.9900	C22—H22B	0.9800
C10—H10B	0.9900	C22—H22C	0.9800
C11—C12	1.534 (3)		
			
O2—S1—O3	118.15 (6)	C13—C12—C11	106.26 (14)
O2—S1—C1	110.68 (6)	C13—C12—H12A	110.5
O3—S1—C1	107.26 (6)	C11—C12—H12A	110.5
O2—S1—C20	108.42 (7)	C13—C12—H12B	110.5
O3—S1—C20	107.92 (7)	C11—C12—H12B	110.5
C1—S1—C20	103.38 (7)	H12A—C12—H12B	108.7
C8—O1—C7	107.06 (10)	C9—C13—C12	104.50 (13)
C8—C1—C2	107.48 (11)	C9—C13—H13A	110.9
C8—C1—S1	127.76 (10)	C12—C13—H13A	110.9
C2—C1—S1	123.69 (10)	C9—C13—H13B	110.9
C7—C2—C3	119.27 (12)	C12—C13—H13B	110.9
C7—C2—C1	104.57 (12)	H13A—C13—H13B	108.9
C3—C2—C1	136.16 (12)	C15—C14—C19	119.42 (13)
C4—C3—C2	118.62 (13)	C15—C14—C8	121.46 (13)
C4—C3—H3	120.7	C19—C14—C8	118.93 (13)
C2—C3—H3	120.7	C16—C15—C14	120.27 (13)
C3—C4—C5	119.61 (13)	C16—C15—H15	119.9
C3—C4—C9	121.76 (13)	C14—C15—H15	119.9
C5—C4—C9	118.61 (12)	C17—C16—C15	118.14 (14)
C6—C5—C4	122.97 (13)	C17—C16—H16	120.9
C6—C5—H5	118.5	C15—C16—H16	120.9
C4—C5—H5	118.5	F1—C17—C18	118.51 (14)
C7—C6—C5	115.49 (13)	F1—C17—C16	117.95 (14)
C7—C6—H6	122.3	C18—C17—C16	123.54 (14)
C5—C6—H6	122.3	C17—C18—C19	117.95 (14)
O1—C7—C6	125.26 (13)	C17—C18—H18	121.0
O1—C7—C2	110.72 (11)	C19—C18—H18	121.0
C6—C7—C2	124.02 (13)	C18—C19—C14	120.62 (14)
C1—C8—O1	110.16 (12)	C18—C19—H19	119.7
C1—C8—C14	136.71 (12)	C14—C19—H19	119.7
O1—C8—C14	113.13 (11)	C21—C20—C22	112.09 (14)
C4—C9—C13	117.50 (13)	C21—C20—S1	110.77 (11)
C4—C9—C10	114.43 (12)	C22—C20—S1	108.30 (11)
C13—C9—C10	101.96 (14)	C21—C20—H20	108.5
C4—C9—H9	107.5	C22—C20—H20	108.5
C13—C9—H9	107.5	S1—C20—H20	108.5
C10—C9—H9	107.5	C20—C21—H21A	109.5
C11—C10—C9	104.54 (13)	C20—C21—H21B	109.5
C11—C10—H10A	110.8	H21A—C21—H21B	109.5
C9—C10—H10A	110.8	C20—C21—H21C	109.5
C11—C10—H10B	110.8	H21A—C21—H21C	109.5
C9—C10—H10B	110.8	H21B—C21—H21C	109.5
H10A—C10—H10B	108.9	C20—C22—H22A	109.5
C10—C11—C12	105.45 (14)	C20—C22—H22B	109.5
C10—C11—H11A	110.7	H22A—C22—H22B	109.5
C12—C11—H11A	110.7	C20—C22—H22C	109.5
C10—C11—H11B	110.7	H22A—C22—H22C	109.5
C12—C11—H11B	110.7	H22B—C22—H22C	109.5
H11A—C11—H11B	108.8		
			
O2—S1—C1—C8	−26.55 (15)	C3—C4—C9—C13	−43.8 (2)
O3—S1—C1—C8	−156.74 (12)	C5—C4—C9—C13	138.05 (15)
C20—S1—C1—C8	89.36 (14)	C3—C4—C9—C10	75.76 (18)
O2—S1—C1—C2	166.81 (11)	C5—C4—C9—C10	−102.40 (16)
O3—S1—C1—C2	36.62 (13)	C4—C9—C10—C11	−168.08 (13)
C20—S1—C1—C2	−77.28 (12)	C13—C9—C10—C11	−40.14 (16)
C8—C1—C2—C7	−0.33 (15)	C9—C10—C11—C12	25.31 (18)
S1—C1—C2—C7	168.63 (10)	C10—C11—C12—C13	−0.8 (2)
C8—C1—C2—C3	179.94 (15)	C4—C9—C13—C12	165.45 (14)
S1—C1—C2—C3	−11.1 (2)	C10—C9—C13—C12	39.50 (17)
C7—C2—C3—C4	−1.0 (2)	C11—C12—C13—C9	−24.25 (19)
C1—C2—C3—C4	178.68 (15)	C1—C8—C14—C15	45.9 (2)
C2—C3—C4—C5	0.4 (2)	O1—C8—C14—C15	−133.59 (13)
C2—C3—C4—C9	−177.73 (13)	C1—C8—C14—C19	−139.22 (17)
C3—C4—C5—C6	0.4 (2)	O1—C8—C14—C19	41.31 (17)
C9—C4—C5—C6	178.60 (13)	C19—C14—C15—C16	−2.3 (2)
C4—C5—C6—C7	−0.5 (2)	C8—C14—C15—C16	172.62 (13)
C8—O1—C7—C6	178.93 (13)	C14—C15—C16—C17	1.3 (2)
C8—O1—C7—C2	−0.37 (15)	C15—C16—C17—F1	−178.20 (13)
C5—C6—C7—O1	−179.33 (13)	C15—C16—C17—C18	1.0 (2)
C5—C6—C7—C2	−0.1 (2)	F1—C17—C18—C19	176.99 (14)
C3—C2—C7—O1	−179.78 (12)	C16—C17—C18—C19	−2.2 (3)
C1—C2—C7—O1	0.43 (15)	C17—C18—C19—C14	1.2 (2)
C3—C2—C7—C6	0.9 (2)	C15—C14—C19—C18	1.0 (2)
C1—C2—C7—C6	−178.88 (13)	C8—C14—C19—C18	−173.99 (14)
C2—C1—C8—O1	0.11 (15)	O2—S1—C20—C21	51.63 (13)
S1—C1—C8—O1	−168.26 (10)	O3—S1—C20—C21	−179.29 (12)
C2—C1—C8—C14	−179.37 (15)	C1—S1—C20—C21	−65.87 (13)
S1—C1—C8—C14	12.3 (3)	O2—S1—C20—C22	−71.66 (12)
C7—O1—C8—C1	0.15 (15)	O3—S1—C20—C22	57.41 (12)
C7—O1—C8—C14	179.77 (11)	C1—S1—C20—C22	170.83 (11)

### Hydrogen-bond geometry (Å, º)

Cg1 is the centroid of the C2–C7 benzene ring.

**Table:**

*D*—H···*A*	*D*—H	H···*A*	*D*···*A*	*D*—H···*A*
C6—H6···O2^i^	0.95	2.60	3.3341 (17)	135
C18—H18···O3^ii^	0.95	2.48	3.2932 (19)	144
C21—H21*A*···*Cg*1^iii^	0.98	2.71	3.693 (2)	177

Symmetry codes: (i) *x*−1, *y*, *z*; (ii) −*x*+3/2, *y*−1/2, −*z*+1/2; (iii) −*x*+1, −*y*+1, −*z*.
